# Strategies for modern biomarker and drug development in oncology

**DOI:** 10.1186/s13045-014-0070-8

**Published:** 2014-10-03

**Authors:** Alan D Smith, Desam Roda, Timothy A Yap

**Affiliations:** Drug Development Unit, Royal Marsden NHS Foundation Trust, Division of Clinical Studies, The Institute of Cancer Research, Downs Road, Sutton SM2 5PT, Surrey, UK

**Keywords:** Biomarkers, Drug development, Targeted therapies

## Abstract

Technological advancements in the molecular characterization of cancers have enabled researchers to identify an increasing number of key molecular drivers of cancer progression. These discoveries have led to multiple novel anticancer therapeutics, and clinical benefit in selected patient populations. Despite this, the identification of clinically relevant predictive biomarkers of response continues to lag behind. In this review, we discuss strategies for the molecular characterization of cancers and the importance of biomarkers for the development of novel antitumor therapeutics. We also review critical successes and failures in oncology, and detail the lessons learnt, which may aid in the acceleration of anticancer drug development and biomarker discovery.

**Electronic supplementary material:**

The online version of this article (doi:10.1186/s13045-014-0070-8) contains supplementary material, which is available to authorized users.

## Introduction

The process of novel drug development from first-in-human studies to registration phase III clinical trials is associated with an unacceptably high attrition rate [1]. Reversing such alarming trends requires rational patient and drug selection to achieve precision medicine in clinical studies [2]. The recognition that intracellular processes drive multiple hallmarks of cancer, including angiogenesis, apoptosis, invasion and metastasis, has highlighted the potential to affect oncogenesis and cancer progression by manipulating these critical processes at a molecular level [3]. Sequencing the cancer genome is a vital component to understanding the molecular basis of cancer; for example, tumor sequencing undertaken at an individual patient level can be utilized to identify specific molecular dependencies and vulnerabilities that may be targeted with antitumor therapies. The BCR-ABL translocation product in chronic myelogenous leukemia (CML), the anaplastic lymphoma kinase (ALK) mutation in lung cancer and the *BRAF* V600E mutation in melanoma are prime examples of specific subsets of cancers that are exquisitely sensitive to rationally selected molecularly targeted antitumor agents [4]–[6].

The Pharmacologic Audit Trail (PhAT) is a drug development framework that can be used to link biomarkers for rational decision-making in early phase clinical trials of novel antitumor therapeutics [7],[8]. The PhAT incorporates a step-wise process, starting with the identification of patients who possess a tumor associated with a specific predictive biomarker that may predict for antitumor response to a particular therapy. While on treatment, pharmacokinetic (PK) profiling and measurement of target and pathway modulation with pharmacodynamic (PD) biomarkers can then be used to ensure active drug exposures are achieved with adequate target engagement [9]. Intermediate endpoint biomarkers may also be used to assess for early signals of clinical response, with the assessment of various biomarkers indicative of resistance mechanisms on disease progression where appropriate [10],[11]. In recent years, a number of molecularly targeted agents have been developed using such strategies that illustrate the importance of a rational approach to drug development. We will discuss strategies for the molecular characterization of patients, and the importance of utilizing different biomarkers in the multistep drug development process. Finally, we will detail key examples that have transformed the landscape of anti-cancer therapeutics, as well as the efforts made in associated biomarker development relevant to these examples.

### Strategies for molecular characterization of patients

In the early 1990s, the first human genome sequenced cost more than $2 billion and took a decade to complete [12]. Novel technologies have seen both processing times and costs fall significantly, such that we are now able to sequence the entire genome in greater detail with improved precision and accuracy [13]. These advances now need to be exploited so as to accelerate oncological drug development and to optimize patient benefit. Such technologies need to be utilized to identify cancers that are more likely to respond to antitumor molecularly targeted agents by exploiting specific dependencies and vulnerabilities through the use of rational clinical trials [14]. Such an approach has the potential to reduce the number and size of large and costly “one-size-fits-all” Phase III trials, as well as the high level of late-phase drug attrition. A refined understanding of underlying tumor biology would ultimately lead to such a discovery through the interrogation of cancer genetic blueprints, for example through DNA sequencing. Commonly employed methods of DNA sequencing may involve genome-wide single nucleotide polymorphism (SNP) microarrays, detection of structural and chromosomal variations, gene-specific Sanger sequencing, and whole genome (WGS) or whole exome sequencing (WES) [15].

### SNP Genotyping

Measuring genetic variation in single nucleotides (SNP genotyping) may potentially identify mutations in genes that have functional consequences. The Affymetrix and Illumina platforms are examples of genome wide SNP genotyping that use hybridization and enzyme-based techniques [16]. Another example is the Sequenom MassARRAY platform, which uses mass spectrometry to detect the mass of the SNP allele extension, rather than a fluorescing molecule, and may not be as useful for whole genome scanning [17]. Overall, SNP genotyping provides a rapid and relatively cost-efficient method to assess the cancer genome for a number of known genetic mutations [18]. One of the major limitations of this technology is the inability to identify non-SNP mutations of interest.

### Next generation sequencing

First generation sequencing (Sanger sequencing) is the original form of WGS DNA sequencing, and allows for long read lengths and high accuracy. However, it may be costly and is low-throughput. Therefore, despite improvements in the process, it has largely been supplanted by next-generation sequencing (NGS) [18]. NGS with WES or WGS has gained favor because it uses massively parallel sequencing assays to interrogate DNA coding regions or the entire euchromatic genome, respectively, resulting in higher throughput. NGS generally involves DNA fragmentation, clonal amplification using polymerase chain reaction (PCR) and sequencing via cyclic enzyme-driven identification of sequential nucleotides, before reconstruction of the original sample is performed using software that aligns overlapping reads from each fragment [18]–[20].

Targeted sequencing is a strategy that has been employed to improve time to acquisition of results and reduction of costs. This process typically involves prior identification of specific genes of interest, followed by targeted exon sequencing using the relevant DNA arrays [21]. Another method, targeted comparative genomic hybridization (CGH), may be used to identify specific gene deletions and duplications [22].

Despite these advancements, there continue to be limitations of NGS rooted in the methodology. Most NGS methods involve amplification of DNA strands, followed by the addition of labeled-bases that can be incorporated into the newly forming fragment by DNA polymerase. The DNA base solvent is then washed out and imaging is used to identify the base incorporated. Repetition of this process is limited by a number of issues, including short read-length which can result in lower accuracy, complex sample preparation, need for amplification, prolonged duration to results, significant data storage, costs and interpretation requirements [18]. Novel third generation technologies, such as PacBio RS and Ion Torrent PGM, have sought to improve on these limitations [23]. Strategies such as single molecule real-time sequencing (SMRTS) developed by Pacific Biosciences directly observes a single molecule of DNA polymerase as it synthesizes DNA, minimizing the need for reagents, eliminating time-consuming washing and scanning steps, and accelerating time to results. Tunneling and transmission electron microscopy directly images the DNA and chemically identifies atoms in nucleotide molecules. This method is currently in development, and promises to increase read lengths at low costs. Another method of third generation sequencing involves DNA sequencing using nanopores. This technology relies on membranes that allow the passage of DNA molecules or nucleotides through holes, and detects their passage by changes in electrical current or optical signals [23].

### Biomarkers for successful drug development

Historically, clinical trials in drug development have employed a toxicity-driven approach to reach a predefined `toxicity ceiling’, maximizing drug exposure with the assumption that this will also maximize antitumor effects. As cancer medicine becomes more sophisticated, the development of novel molecularly targeted agents has required researchers to refine this approach for a number of reasons. Firstly, targeted agents are designed to block a specific molecule or intracellular pathway and therefore often have a limited toxicity profile relative to cytotoxic chemotherapies. As a result, several of these therapies never reach a `toxicity ceiling’ or maximum tolerated dose (MTD) in classically designed dose-escalation trials. A greater understanding of the intracellular pathways integral to cancer cells will also facilitate the determination of PD effects by measuring the activity of downstream markers and alternate pathways. This allows for the potential to tailor dose and dosing schedule to PD drug effects, rather than toxicities. Interest in developing methods to evaluate treatment efficacy earlier in the treatment course has fueled the investigation of novel biomarker assays as possible intermediate endpoint biomarkers of response.

Promising novel biomarkers should be systematically assessed, both retrospectively and prospectively [24]. Ultimately, we foresee the goal of developing multiple biomarkers (i.e. predictive, pharmacodynamic, pharmacokinetic, pharmacogenomic and intermediate endpoint biomarkers) for incorporation within intelligent, hypothesis-driven early phase clinical trials to combine with robust outcome data. The discovery and evaluation of any novel biomarkers will ideally be certified to Clinical Laboratory Improvement Amendments (CLIA) and Good Clinical Laboratory Practice (GCLP) standards, so as to ensure accuracy and reproducibility of laboratory procedures. In this section, we highlight and discuss the critical biomarkers that will be vital for the successful development of novel molecularly targeted therapeutics.

### Predictive biomarkers

Predictive biomarkers indicate the likelihood of response to a specific antitumor therapy. Such assays should be scientifically sound, have preclinically validated methodologies, and have been clinically proven in prospective randomised trials to robustly and reproducibly predict antitumor efficacy in the applied patient population [9]. Predictive biomarkers include both tumor-specific and surrogate biomarkers, and are crucial to accelerating the drug development process. For example, ERBB2 (HER2) is a cellular transmembrane tyrosine kinase encoded by the *ERBB2* gene. HER2 overexpression or amplification in breast cancers is a useful biomarker that has been critical for the identification of patients who are likely to respond to HER2 targeting drugs, thereby enabling the development of trastuzumab [25],[26], pertuzumab [27], trastuzumab-DM1 [28],[29] and lapatinib [30]. Another well-established predictive biomarker is the oncogenic *BCR-ABL* gene fusion, which predicts for antitumor responses to the tyrosine kinase inhibitor imatinib in chronic myelogenous leukemia [31]. More recently, biomarkers that have been used to enrich or predict for sensitivity to a targeted agent include *BRCA1* and *BRCA2* mutations to the PARP inhibitor olaparib [32]-[35]; *EML4-ALK* fusions to the ALK/MET inhibitor crizotinib [5]; V600E *BRAF* mutation to the BRAF inhibitor vemurafenib [36]; *EGFR* wild-type metastatic colorectal cancer to the EGFR-targeted antibodies panitumumab [37] and cetuximab [38]; and *EGFR* mutant advanced non-small cell lung cancer (NSCLC) to the small molecule inhibitors gefitinib [39] and erlotinib [40],[41].

The term `enrichment biomarker’ has been used to describe biomarkers with strong scientific rationale and preclinical evidence for antitumor response, but which lack clinical validation [7]. Such enrichment biomarkers currently in clinical trials may of course be clinically qualified and become predictive biomarkers in the future. Examples include PTEN loss or *PIK3CA* mutations for PI3K-Akt-mTOR pathway inhibitors (NCT01458067; NCT01449370); *RAS* mutations for the combination of MAPK and PI3K pathway inhibitors (NCT01449058) and IGF mutations with IGF-1R antibodies (NCT01403974; NCT01562899). Different biomarker panels, such as the TruSeq Amplicon – Cancer Panel (TSACP), have been developed to facilitate the identification of relevant biomarkers (i.e. genetic mutations) for research, and can be a useful point-of-care test [42].

### Pharmacodynamics, pharmacokinetics and pharmacogenomics

The use of molecularly targeted agents has necessitated PD biomarkers, which indicate drug effects on the target, pathway and downstream cellular processes [7],[43]. Preclinical studies to establish and evaluate PD biomarkers are therefore essential for the development of novel antitumor therapeutics. Similarly, PK profiling is crucial to ensure active drug exposures and to establish PK-PD drug profiles and toxicity relationships [9]. This information can then be used to direct Phase I studies by providing PK and PD thresholds to target. Use of fresh tumor tissue still remains the gold standard for biomarker evaluation; however obtaining normal tissue such as platelet-rich plasma, peripheral blood mononuclear cells, hair follicles and skin is relative less invasive and can be sampled serially, thereby minimizing inter- and intra-patient variability [44]. PK and PD variability between patients following fixed doses of targeted therapies may also be affected by individual pharmacogenomic factors. Such genetic variability between hosts may impact the expression or function of proteins that metabolize the drug or may affect the drug target itself, thereby affecting treatment efficacy and toxicity. The current clinical practice is to dose adjust for patients who experience unacceptable toxicities; however, such practice will mean that a substantial proportion of patients are inevitably undertreated unless their dose is escalated.

### Intermediate endpoint biomarkers

Intermediate endpoint (surrogate) biomarkers indicate treatment efficacy at an earlier time point than the primary endpoint of the study, and can therefore substitute for the clinical primary endpoint [45]. Established surrogate biomarkers can therefore accelerate drug approval, facilitate earlier decisions about treatment efficacy and mitigate costs and morbidity related to treatment. Numerous biomarkers have been studied for surrogacy, however trials evaluating their validity are often poorly reproducible, inaccurate, inconsistently applied or only loosely associated with survival [46],[47]. Changes in tumor markers, such as PSA and CA125, continue to be used as surrogate biomarkers, however the data remains controversial as to how predictive they are for survival [46],[47]. Progression-free survival (PFS) is also often used as a surrogate for overall survival, however, this remains controversial in many cancers, particularly in the era of targeted therapies [48]–[50]. More recently, circulating tumor cells (CTCs) have been evaluated as a surrogate biomarker in a number of cancers, including castration-resistant prostate cancer (CRPC), lung, breast and colon cancer [51]-[59]. Circulating plasma DNA also appears to have promising utility as a biomarker, demonstrating correlation with tumor behaviour and changes in cancer burden in malignancies such as breast, lung, gastrointestinal stromal tumors and ovarian cancers [60]–[64]. Other studies have documented detectable levels of circulating plasma DNA in pancreatic, colorectal, bladder, gastroesophageal, melanoma, hepatocellular, and head and neck cancers [65]. Ultimately, better preclinical models may be helpful in deciphering the relevant molecular mechanisms of response and resistance. Patient-derived tumor xenografts (PDX) and genetically-engineered mouse models (GEMM) are two model systems that can be used to study surrogate markers in the laboratory [66]. Recently, further progress has been made in the use of PDX models for developing biomarker-driven hypotheses that can be tested in the clinic to identify patients that may benefit from a therapeutic intervention [67].

### Proteomics and metabolomics

The cancer proteome (i.e. the complete set of proteins expressed by the cancer) and the cancer metabolome (i.e. the entire set of small molecule metabolites produced by the cancer) may also be informative [68]. Methods such as mass spectrometry, electrophoresis and protein microarrays can be used to profile metabolomic and proteomic signatures, and identify molecules that are differentially expressed in certain cancers [68]–[71]. Mass spectrometry can also be used for `metabolic phenotyping’ by mapping the interconnected networks of biochemical pathways, which may lead to the identification of candidate biomarkers [72]. Low abundance proteins, for example in a single cell type, can be evaluated using deep proteomics methods, such as liquid chromatography and high-resolution mass spectrometry [73].

### Modern functional imaging biomarkers

Well-established imaging modalities such as computed tomography (CT) and magnetic resonance imaging (MRI) are important techniques used to structurally evaluate tumor growth and drug efficacy in clinical practice. However, they are not able to functionally assess lesions, nor do they address any specific molecular processes within the tumor. The availability of novel functional imaging probes may enable the assessment and monitoring of molecular pathways involved in a range of cellular processes, including angiogenesis, metabolism, cell proliferation, infiltration, metastasis and apoptosis [74]. Functional imaging techniques such as dynamic contrast-enhanced MRI (DCE MRI), diffusion-weighted imaging MRI (DWI MRI), 18 F-fluorodeoxyglucose positron emission tomography (FDG-PET), and magnetic resonance spectroscopy (MRS) are frequently employed modalities that can measure the relevant cancer-specific molecules and signaling pathways [43],[75]. For instance, PET tracers can be used quantitatively to measure markers of cellular proliferation, cell hypoxia and apoptosis [76]. Dynamic image acquisition and compartmental modelling can also be used with PET/CT to assess tumor perfusion, tracer extraction and tissue metabolism [77]. In addition, new methods of MRI have enabled dynamic, functional and metabolic assessments of changes in the tumor vascular network with perfusion or permeability imaging, such as dynamic contrast-enhanced (DCE) MRI, or the tumor microenvironment with diffusion-weighted imaging (DWI) [78]–[80]. Such methods allow rapid non-invasive evaluation of tumor response and mechanisms of drug action. Nevertheless, issues including high costs and variability in techniques between institutions and MRI platforms have limited the routine application of functional MRI readouts as a valid biomarker [76].

### Lessons learnt from successes in drug development

**Figure 1:**
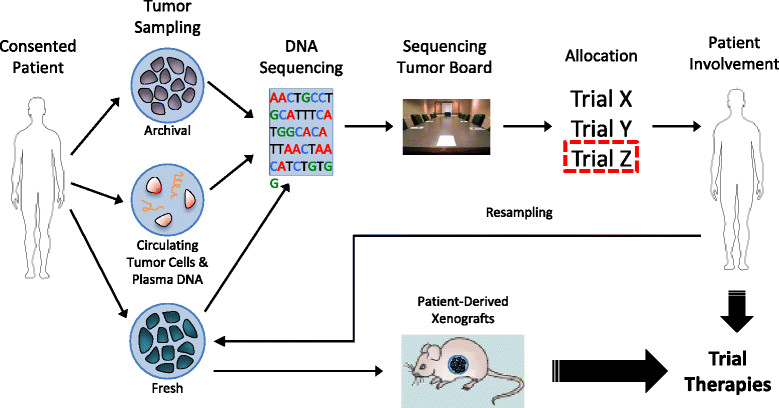
**Translation of sequencing into Clinical Oncology & Research.** Patients start with informed consent to access their archived tumor sample. When applicable, circulating tumor cells and plasma DNA are sampled; as well as accessible fresh tumor. Tumor DNA is then purified and sequenced, and the results are presented at a tumor board of experts, where the patient is then allocated to an appropriate trial based on the results. Fresh tumor can also be grafted into mice to determine if a theraphy is effective, before giving to the patient. Tumor resampling after progression on theraphy is essential to identifying the mechanism of resistance and a new treatment strategy.

**Figure 2:**
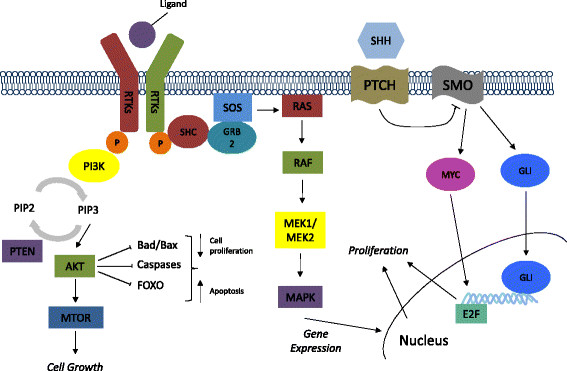
**Molecular pathways in cancer progression.** This figure identifies some of the common pathways involved in cancer cell growth and proliferation. Proteins such as Receptor Tyrosine Kinases (RTKs) (i.e. EGFR, HER2, VEGFR, PDGFR, IGFR, KIT) PI3K, AKT, RAF, MEK, and SHH represent some of the drug targets in precision medicine.

There have been several examples of successful monoclonal antibody and small molecule drug development programs in the field of oncology over the past decade (Table 1). To date, multiple targeted therapies have been approved by the US FDA, with many more currently in different phases of clinical trial testing [81]. In this section, we detail key examples that have paved the way for the development of other novel antitumor agents, and discuss efforts and progress made in biomarker discovery for such drugs. Figure 1 illustrates a potential patient pathway in the clinic for rational biomarker and clinical drug development. Figure 2 highlights key pathways that are targeted by novel anti-cancer therapeutics, as discussed below.

**Table 1:** Identified biomarkers and their relevant drugs in selected cancers*

Biomarker	Drug	Drug action	Cancer type (Survival benefit)
**ALK**	Ceritinib	Tyrosine kinase inhibitor of ALK	Lung cancer
	Crizotinib	Tyrosine kinase inhibitor of ALK	Lung cancer
**BRAF (V600E)**	Dabrafenib	Inhibits B-RAF protein	Melanoma
	Trametinib	Inhibits MEK1 and MEK2 growth factor-mediated signaling	Melanoma
	Vemurafenib	Small molecule inhibitor of BRAF (V600E) kinase	Melanoma
**CTLA-4**	Ipilimumab	Monoclonal antibody directed against CTLA-4, enhancing T-cell activation	Melanoma
**EGFR**	Afatinib	Irreversibly inhibits EGFR, HER2, HER4, mutant EGFR (exon 19, 21)	Lung cancer
	Cetuximab	Recombinant, chimeric, monoclonal antibody directed against EGFR	Colorectal cancer, SCCHN**
	Erlotinib	Reversible tyrosine kinase inhibitor of EGFR	Lung cancer, Pancreatic cancer
	Gefinitib	Tyrosine kinase inhibitor of EGFR	Lung cancer
	Panitumumab	Humanized monoclonal antibody directed against EGFR	Colorectal cancer
**HER2**	Lapatinib	Reversible tyrosine kinase inhibitor of EGFR, HER2	Breast cancer
	Pertuzumab	Recombinant, humanized, monoclonal antibody preventing HER2 dimerization	Breast cancer
	Trastuzumab	Recombinant, humanized, monoclonal antibody directed against HER2	Breast cancer, Gastric cancer
	Trastuzumab-mertansine (T-DM1)	Antibody-drug conjugate consisting of trastuzumab conjugated to DM1, which binds tubulin and disrupts microtubule assembly/disassembly dynamics	Breast cancer
**KIT**	Imatinib	Tyrosine kinase inhibitor of c-kit	GIST***
	Sunitinib	Tyrosine kinase inhibitor of VEGFR2, PDGFRb and c-KIT	GIST***
**MEK**	Trametinib	MEK1/2 inhibitor	Melanoma

*Solid tumor malignancies.

**SCCHN = squamous cell cancers of the head and neck.

***GIST = gastrointestinal stromal tumors.

### Monoclonal antibodies

#### Angiogenesis inhibitors

Bevacizumab is a humanized monoclonal antibody against vascular endothelial growth factor A (VEGF-A), which is a critical factor required for the growth of blood vessels in tumors [82]. It was the first anti-angiogenic drug approved for the treatment of patients with advanced colorectal cancer [83]. However, despite its broad clinical activity, no definitive predictive biomarkers of antitumor response have been identified, despite multiple large high profile clinical studies in this area [84]. For example, Cameron and co-workers undertook a phase III trial assessing the role of bevacizumab in patients with resected triple-negative breast cancer [85]. In this study, 2591 patients were randomly allocated to four or more cycles of anthracycline-based or taxane-based chemotherapy with or without bevacizumab. The primary endpoint of progression-free survival was however not met and importantly grade 3 or worse toxicity was more common with the bevacizumab arm [85],[86].

In addition, bevacizumab is the only antiangiogenic drug approved by the FDA for the first-line management of NSCLC and HER2-negative breast cancer, and second-line treatment of glioblastoma, and metastatic renal cell carcinoma [87]. Bevacizumab efficacy for advanced lung cancer was recently demonstrated in a number of clinical trials, which demonstrated improved OS and PFS, albeit only by a few months [88]-[90].

Recently, Lambrechts and colleagues investigated novel promising biomarkers of response for bevacizumab treatment [91]. While VEGF-A isoforms and modified expression of VEGFRs (VEGFR1 and NRP1) appeared to be good candidates, these biomarkers demonstrated a lack of consistency across studies in multiple cancer types on retrospective analysis [91]. The ongoing MERIDIAN trial in metastatic breast tumors will evaluate the impact of bevacizumab treatment in patients stratified by plasma short VEGFA isoforms (NCT01663727).

### Epithelial growth factor receptor inhibitors

The EGFR pathway is another commonly targeted signaling cascade in antibody therapeutics. Cetuximab is an IgG1 anti-EGFR antibody targeted against the extracellular domain of EGFR and is approved for use in advanced colorectal cancer and squamous cell carcinoma of the head and neck (SCCHN) [38],[92]-[94]. In comparison, panitumumab is a fully humanized IgG2 monoclonal antibody, which also targets EGFR and is approved for use in metastatic colorectal cancer [95],[96].

It is now clear that *KRAS* mutations are a negative predictive indicator of response to anti-EGFR therapy in view of downstream pathway activation [97]-[99]. However, the use of EGFR expression testing is still controversial since the large phase III CRYSTAL trial showed no correlation between treatment response and immunohistochemical EGFR determination [38].

In advanced SCCHN, although a survival advantage was demonstrated with cetuximab plus concurrent chemotherapy or radiotherapy, to date, no predictive biomarkers of response have yet been identified [94]. Vermorken and co-workers recently published the SPECTRUM trial results, where chemotherapy plus panitumumab were compared to chemotherapy alone in patients with relapsed SCCHN [100]. A subgroup analysis revealed that p16-negative tumors treated with anti-EGFR therapy presented a significantly higher survival benefit compared to p16-positive tumors. Despite this being a retrospective subgroup analysis, p16 status appears to be a relevant biomarker for patients with SCCHN, and should be explored prospectively in the future [101].

### Human epidermal growth factor receptor 2 inhibitors

The development of anti-HER2 targeting agents has dramatically altered the management of HER2-positive breast cancer and is one of the first successes of molecularly targeted therapies in oncology. Currently, three antibodies are approved for the treatment of HER2-positive breast cancer. These include trastuzumab, a monoclonal IgG1-class humanized murine antibody directed against HER2; pertuzumab, a monoclonal IgG1-class antibody that inhibits the dimerization of HER2 with other HER receptors; and trastuzumab emtansine (T-DM1), an antibody drug conjugate [102].

Well-recognized predictive biomarkers of response to anti-HER2 therapy are HER2 overexpression (3+) measured by standardized immunohistochemistry (IHC) methods or gene amplification demonstrated by fluorescent in situ hybridization (FISH) [103],[104]. In the HER2-positive population, trastuzumab has moderate monotherapy antitumor effects. However, such efficacy is enhanced in combination with conventional chemotherapy, achieving a higher rate of objective responses (50% vs. 32%, P < 0.001), longer duration of response (median 9.1 vs. 6.1 months; P < 0.001) and longer survival (median survival 25.1 vs. 20.3 months; P = 0.01) [26],[105],[106].

Compared to trastuzumab, pertuzumab is a monoclonal antibody that sterically blocks the dimerization of HER2 with HER1, 3 and 4 and binds to a different HER2 epitope [107], resulting in synergistic antitumor effects when combined with trastuzumab. Pertuzumab treatment was approved for use in combination with trastuzumab and standard chemotherapy in HER2-positive metastatic breast cancer after showing significantly prolonged progression-free survival (18.5 vs 12.4 months; P = 0,001), importantly with no increase in cardiac toxic effects [108]. In the adjuvant setting, the addition of trastuzumab to cytotoxic chemotherapy resulted in a remarkable 50% reduction in disease recurrence in a preselected HER2+ breast cancer patient population [109],[110].

A retrospective study published by Paik and colleagues [111] showed that HER2-positive patients could also benefit from adjuvant trastuzumab. They identified 174 patients from the NSABP B31 study, who lacked HER2 gene amplification despite being originally reported as HER2-positive. Surprisingly, analysis of outcome data revealed that these HER2-positive patients benefited as much from adjuvant trastuzumab as did patients whose tumors displayed HER2 gene amplification. These findings are now being studied prospectively in a randomized phase III trial (NSABP B47; NCT01275677).

### Antibody-drug conjugates

The ability to deliver cytotoxic chemotherapeutic agents directly to cancer cells with targeted conjugates has been an area of interest for some time, although its development has been technically challenging to develop until recent times. T-DM1 is an antibody-drug conjugate consisting of trastuzumab (T) linked to the small molecule cytotoxic payload mertansine (DM1) [112]. T-DM1 was recently approved for the treatment of patients with HER2-positive metastatic breast cancer that has progressed on prior trastuzumab and taxane chemotherapy [113]. This followed the EMILIA phase III trial, which showed that T-DM1 produced a high percentage of responses and significantly improved PFS and OS compared to standard therapies for advanced HER2-positive breast cancers. Ongoing phase III trials (MARIANNE and THERESA) are testing this drug in different settings within the breast cancer population. (NCT01120184 and NCT01419197).

Recently, Phillips and co-workers [114] explored, for the first time, the dual targeting of HER2-positive cancer with TDM1 and Pertuzumab. They explored this dual combination initially in cultured tumor cells, followed by mouse xenografts and finally humans in a single-arm phase Ib/II study. This combination showed an encouraging safety and tolerability profile with preliminary evidence of efficacy. Interestingly, investigators observed that the presence of the HER3 ligand, heregulin (NRG-1β), reduced the cytotoxic activity of T-DM1 *in vitro*; and that such effects were reversed by the addition of pertuzumab. This indicates a potential resistance mechanism to HER2-targeted therapies.

In hematological cancers, the antibody drug conjugate Brentuximab-vedotin has already been approved for relapsed Hodgkin lymphoma and systemic anaplastic large cell lymphoma, and other similar compounds are also currently in development[115].

### Small molecule inhibitors

#### Small molecule angiogenesis inhibitors

Sunitinib, pazopanib and sorafenib are all multikinase inhibitors that block angiongenesis targets (among others) and have gained FDA-approval in the last five years for renal cell carcinoma (RCC) and hepatocellular carcinoma (HCC). Although there are no validated biomarkers for targeted therapies in advanced RCC or HCC, different groups have undertaken retrospective studies on sunitinib, pazopanib, and sorafenib for potential biomarkers in order to understand the interpatient variability in clinical benefit [116]-[120]. Previous studies in metastatic RCC have demonstrated a potential correlation between baseline soluble protein levels and efficacy for sunitinib [116], and sorafenib [121]. In addition, genetic variability, such as germline single-nucleotide polymorphisms (SNPs) in VEGF-related genes (e.g.VEGF-A and VEGFR3), has been investigated as a potential predictive biomarker for such antiangiogenic agents [116],[121].

### EGFR inhibitors

The approval of tyrosine kinase inhibitors (TKIs) such as erlotinib and gefitinib was a key milestone for the treatment of NSCLC, by presenting a model for targeted therapy development and genetic profiling of this disease.

Retrospective analysis and subsequent prospective clinical trials have demonstrated that *EGFR* mutant lung adenocarcinoma benefits from anti-EGFR therapy, although some controversy still exists regarding how best to select these patients. Two recent studies reviewed published data regarding this topic; Lee and colleagues in their meta-analysis concluded that EGFR mutations are a predictive biomarker of PFS benefit to anti-EGFR TKIs; Goss and co-workers confirmed this conclusion, and also suggested that an activating mutation predicts a greater likelihood of antitumor response and that there are a proportion of EGFR wild-type patients with NSCLC who will still gain some benefit from this therapy [122],[123].

Second generation EGFR TKIs including afatinib and dacomitinib, bind EGFR irreversibly [124],[125]. In preclinical studies, these drugs overcome resistance via T790M mutation (threonine to methionine at the 790 locus, the most common resistance mechanism), however the concentration required to overcome T790M activity is not achievable in patients [126],[127] due to dose-limiting toxicity related to non-selective inhibition of wild-type EGFR [128]. As a result, third generation EGFR TKIs such as WZ4002 [129], CO1686 [130], AZD9291 [131], and HM61713 [132] are currently in development, and have been designed to target T790M and EGFR TKI-sensitizing mutations more selectively than wild-type EGFR [131]-[134].

A range of strategies combining TKIs with other therapies have recently been explored [135]. Despite favorable preclinical data suggesting an increase in efficacy by combining dual anti-EGFR blockade (e.g. using a combination of anti-EGFR monoclonal antibody and a small molecule inhibitor), clinical results were disappointing [136]. In contrast to patient benefit observed with inhibiting the EGFR pathway with cetuximab in conjunction with cytotoxic chemotherapy in gastrointestinal malignancies, gefitinib and erlotinib showed no clinical benefit and an increase of drug-related toxicities when combined with conventional chemotherapy agents in lung cancer [137],[138].

### ALK inhibitors

The 4-year period from the identification of the oncogenic ALK gene rearrangement (*ALK-* positive) in NSCLC to crizotinib approval was incredibly rapid, and an excellent example of hypothesis-testing, biomarker-driven drug development [139]. Impressive results from phase I and II trials led to FDA approval of the ALK inhibitor crizotinib in ALK-translocated NSCLC in 2011 [5],[140].

Patients with ALK-positive lung cancer comprise only 4-5% of those with NSCLC; however, lung cancer is a highly prevalent disease, and therefore ALK-driven tumors represent a relatively large target population when compared to rare tumors. Most tumors develop resistance to crizotinib therapy after one to two years of treatment, and two studies involving a small series of crizotinib-resistant patients have recently been published [141],[142].

In these studies, secondary ALK mutations were the most common drug-resistance mechanism, present in up to one third of the target population, predominantly affecting the tyrosine kinase domain (L1196M). ALK amplifications were also described in the presence or absence of ALK mutations. Other mechanisms of resistance proposed include the development of *EGFR*, *KIT* and *KRAS* gene aberrations as bypass pathways mediating crizotinib resistance [6],[143]-[146].

### BRAF and MEK inhibitors

In 2011, vemurafenib was approved for the treatment of BRAF-mutant melanoma patients [6]. Dabrafenib has since also been approved as the second BRAF inhibitor to achieve FDA approval for the treatment of advanced melanoma [147]. Although response rates with vemurafenib in patients with V600E *BRAF*-mutated melanoma have been impressive, this inhibitor is associated with a PFS of only 5-7 months, likely due to the development of compensatory mechanisms of resistance described below [148],[149].

Recently, the MEK inhibitor trametinib was also approved by the FDA for advanced melanoma, after showing increased survival compared to chemotherapy in a phase III trial [150]. In addition, MEK inhibitors have shown efficacy in those patients progressing on BRAF inhibitors [151]. Critically, BRAF and MEK inhibitors may have better results when given concomitantly rather than sequentially, with concurrent treatment associated with increased antitumor responses to therapy and a reduction in the severity of toxicities observed [150],[151]. Different phase III trials exploring this combination are ongoing (NCT01689519; NCT01597908; NCT01584648).

Multiple resistance mechanisms have been identified with these drugs, including those secondary to the reactivation of the MAPK pathway (e.g. *BRAF* amplification or mutations, truncations in the BRAF protein, or secondary mutations in *NRAS* and *MEK*), as well as activation of other pathways, such as the PI3K-AKT-mTOR and VEGF signaling pathways [148],[149],[152]-[154]. Multiple combination regimens, based on preclinical data, are now planned or ongoing, such as the combination of BRAF inhibitors with VEGF inhibitors (NCT01495988).

### Other small molecule inhibitors

Imatinib, a 2-phenyl amino pyrimidine derivative, is a tyrosine kinase inhibitor with selectivity against c-KIT, ABL, BCR-Abl, and PDGFR-α. It was coined the “magic bullet” in 2001 when it became the first example of a biomarker-guided therapy that revolutionized the treatment of chronic myeloid leukemia (CML). It was also subsequently approved by the FDA for the first-line treatment of GIST [87].

Despite its clinicopathologic heterogeneity, GIST commonly shares similar oncogenic mutations that involve KIT or PDGFR [155],[156]. Clinical trials have demonstrated benefit with imatinib in unresectable or metastatic GIST [157]-[161], and also in the adjuvant setting for resectable primary tumours [162],[163]. Mutational analysis has a key role in this disease, both to confirm the initial diagnosis, and for the characterization of prognostic and response biomarkers for the administration of molecularly targeted therapies [164]. For example, GIST responds better to imatinib therapy if it harbours a mutation in exon 11, versus tumors with mutations in exon 9 or without any mutations detected [164]-[166]. In addition, the PDGFR-α D842V mutation appears to confer imatinib resistance [166]. Interestingly, while the appearance of certain secondary mutations may be associated with imatinib resistance, they may remain sensitive to other tyrosine kinase inhibitors such as sunitinib, masitinib, nilotinib or dasatinib therapy. For example, imatinib-resistant tumors with a KIT mutation in exon 9 still respond to sunitinib, which was FDA-approved for second-line treatment of advanced GIST in 2006 [167]. The inhibition of KIT activity with sunitinib treatment has also been investigated in GIST [168]. Improved clinical benefit and survival were demonstrated with primary *KIT* wildtype, and *KIT* exon 9 mutations versus *KIT* exon 11 mutations [169]. In addition, secondary *KIT* exon 13 and 14 mutations have been associated with better survival rates compared to *KIT* exon 17 and 18 mutations [169].

Vismodegib is a first-in-class, small-molecule inhibitor of SMO, a key component of the hedgehog pathway. It was approved for the treatment of metastatic or locally advanced basal cell carcinoma (BCC) not suitable for local salvage therapies. The ERIVANCE phase II trial showed promising signs of efficacy with a response rate of 30-43% and a 7.6-month median duration of response [170]-[172]. This is an encouraging example of a rationally-driven study based on the dysregulation of the hedgehog signaling pathway in BCC leading to antitumor activity. In addition, vismodegib has demonstrated patient benefit in those with basal cell nevus syndrome involving germline PTCH1 alterations [173],[174]. There are currently ongoing studies which are exploring new schedules of vismodegib to minimize drug toxicities, and other trials assessing the activity of vismodegib in the adjuvant setting (NCT01815840, NCT01631331, NCT01898598) [175].

### Immunotherapies

In recent years, the field of tumor immunotherapy has taken center stage in oncology drug development. Notable clinical successes include the CTLA-4 inhibitor ipilimumab for melanoma and PD-1/PDL-1 inhibitors [176],[177].

Ipilimumab is an IgG1 monoclonal antibody against the extracellular domain of CTLA-4, which consequently leads to the inhibition of an early point in T-cell activation. In late phase trials, ipilimumab has demonstrated antitumor activity in patients with advanced melanoma [178],[179]. For example, in a randomized first-line phase III trial that enrolled patients with advanced melanoma, the dacarbazine plus ipilimumab combination prolonged overall survival compared to dacarbazine plus placebo. The combination arm was however also associated with a high frequency of Grade 3-4 adverse events [178].

More recently, the clinical development of antibodies against PD-1, such as nivolumab and lambrolizumab (MK3475), as well as PDL-1 inhibitors, such as MDX-1107 or MPDL-3280, have produced impressive antitumor responses, up to 50% [180],[181]. In melanoma patients, it also has a more favorable drug-toxicity profile compared to ipilimumab [179],[182]-[184]. There appear to be complementary mechanisms of action for ipilimumab and the anti-PD-1/anti-PDL-1 antagonists, with recent clinical studies demonstrating that the two agents in combination have an impressive additive activity in patients with advanced melanoma [185]. Kefford and colleagues [181] recently demonstrated that PDL1-positive melanomas were significantly more likely to respond to MK3475 (51%) than PDL1-negative tumors (6%) (p = 0.0012). These patients also had improved progression free survival (12 vs 3 months respectively; p = 0.0004) [181]. Although a similar pattern has been found in lung cancer patients [186], this did not reach statistical significance, and PDL-1 testing is being further evaluated in a number of other cancers [187]-[191]. Interestingly, some PDL-1-negative tumors also respond, underlining the need for more sensitive biomarkers [181].

## Conclusions

The face of oncology is rapidly changing, with the increased use of genetic profiling for the identification of critical targets involved in the hallmarks of cancer. The development of novel therapeutics, particularly those targeting key molecular pathways, will require systematic and rational planning to minimize the treatment of patients with ineffective and potentially toxic drugs. However, the current paradigm of drug development is also economically unsustainable, due to the ever-escalating costs associated with drug attrition from preclinical to clinical studies and large one-size-fits-all phase III trials. We believe that analytically validated and clinically qualified predictive biomarkers of response hold the promise to curbing these issues. We therefore suggest an increased focus on the Pharmacological Audit Trail to discover and validate such biomarkers to accelerate drug development.

In the coming years, as critical cancer drivers are further elucidated, we anticipate the accelerated development of many more targeted monoclonal antibodies and small molecule inhibitors [192],[193]. Also, molecularly targeted antibody-drug conjugates appear to be a promising method of delivering cytotoxic drugs in a targeted manner, thereby optimizing drug exposure to cancer cells while minimizing patient toxicity. In the future, we anticipate that antibody therapeutics will also increasingly involve modifications to the Fc domain in order to trigger the immune system through the activation of T cells and Fcγ-positive accessory cells (e.g. macrophages, dendritic cells, natural killer cells). In addition, new drug designs comprising bi-functional or tri-functional bi-specific antibodies or attenuated single-chain antibodies will be tested. For example, ertumaxomab, a tri-functional bi-specific antibody that targets two different antigen binding sites (anti-Her2 and anti-CD3) and the typical Fc region, was tested in phase I and phase II studies and showed clinical benefit and potent immunological response in HER2-positive breast cancer [19]. Finally, we anticipate that the immunotherapy drug development field will be further expanded, including combination therapies with molecularly targeted therapeutics.

## Authors’ contributions

AS contributed to this article as primary author and researcher, by writing and coordinating the drafting of the manuscript. DR participated in the researching and writing of the manuscript. TY conceived of the review content, and revising by participating as the senior author of the manuscript. All authors read and approved the final manuscript.

## Authors’ original submitted files for images

Below are the links to the authors’ original submitted files for images. Authors’ original file for figure 1 Authors’ original file for figure 2
