# Supplementary figures and images for: Strategies for modern biomarker and drug development in oncology
Source: J Hematol Oncol. 2014 Oct 3;7:70. doi: 10.1186/s13045-014-0070-8 (PMC4189730; doi:10.1186/s13045-014-0070-8)

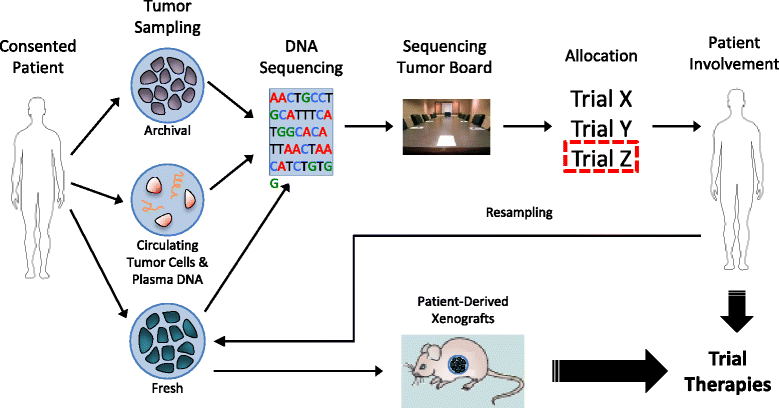

Supplement: Supplementary file 1 — Authors’ original file for figure 1 [file 13045_2014_70_MOESM1_ESM.gif]

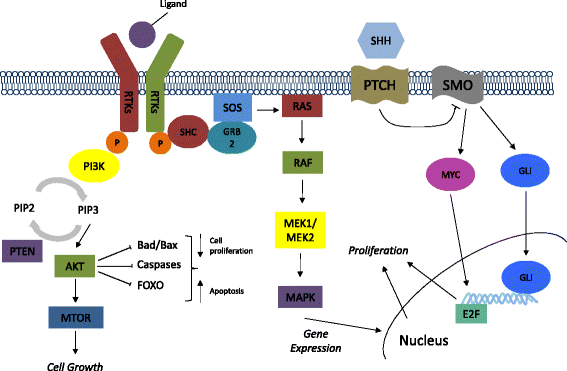

Supplement: Supplementary file 2 — Authors’ original file for figure 2 [file 13045_2014_70_MOESM2_ESM.gif]
